# Supplementary material for: Surveying Parents’ Awareness and Adherence to Influenza Vaccination Recommendations in Children in Southern Italy
Source: Vaccines (Basel). 2022 Aug 11;10(8):1298. doi: 10.3390/vaccines10081298 (PMC9412990; doi:10.3390/vaccines10081298)
Supplement: Supplementary file 1 [file vaccines-10-01298-s001.zip › S1 Sample size calculation.pdf]

Sample size calculation*

The minimum sample size was calculated as follows:

$$n = \frac{Z\frac{\alpha 2}{2}\ P(1-P)}{d^2}$$

where,

P = estimated proportion

d = desired precision

$$n = \frac{1.96\ 0.50(0.50)}{0.025} = 384$$

*Aday, L. A., Cornelius, L. J. (2006). *Designing and conducting health surveys: A comprehensive guide* (3rd ed.). Jossey-Bass.
