# Supplementary material for: Surveying Parents’ Awareness and Adherence to Influenza Vaccination Recommendations in Children in Southern Italy
Source: Vaccines (Basel). 2022 Aug 11;10(8):1298. doi: 10.3390/vaccines10081298 (PMC9412990; doi:10.3390/vaccines10081298)
Supplement: Supplementary file 1 [file vaccines-10-01298-s001.zip › S2 Questionnaire.pdf]

QUESTIONNAIRE

Section A. Socio-demographic and anamnestic characteristics

A1. Gender: □ male □ female □ other, specify ________________

A2. What is your nationality? □ Italian □other nationality, specify__________________

A3. What is your date of birth? **d___/m____/y___**

A4. What is your marital status? □ married □ separated □ divorced □ widowed □ cohabitant □ unmarried □ other, specify _______________

A5. What is your occupation? ____

A6. What is your partner's occupation?(**If applicable**) ___________________________________

A7. What is the highest level of education that you have completed? □ none □ primary school □ middle school □ high school □ university degree □ other, specify ______________________________

A8. What is the highest level of education that your partner completed?(**If applicable**) □none □ primary school □ middle school □ high school □ university degree □ other, specify _______________________

A9. Have you ever received a influenza vaccination? □ no (**Go to A11**) □ yes, when? (**more than one answer allowed**)
□ 2018\19 influenza season □ 2019\20 influenza season □ 2020\21 influenza season □ 2021\22 influenza season

A10. In the hours or days following vaccination, did you have any side effects attributable to the vaccine □ no □ yes, which ones? ____

A11. How many sons/daughters do you have: n. ___ What is your child's date of birth? **d___/m___/y___**

A12. Does your child suffer from chronic diseases? □ no □yes, which ones? □ cardiovascular diseases □ respiratory diseases □ diabetes □ oncohematological diseases □ □ neuromuscular diseases □ other, specify _______________________________________

A13. Have your child been examined by a physician in the last 12 months? □ no
□ yes, primary care physician, how many times?________ □yes, pediatrician, how many times?___________
□ yes, other specialist, specify______________, how many times?________

B. Knowledge regarding influenza and related vaccination

B1. In the following table, please indicate which vaccinations are mandatory or recommended for 6 months-6 years old children:

**Vaccine against**	**yes**	**no**	**uncertain**	**Vaccine against**	**yes**	**no**	**uncertain**
Diphteria/tetanus/pertussis	□	□	□	Poliomyelitis	□	□	□
Hepatitis B	□	□	□	Rotavirus	□	□	□
Meningococcus A,C,W,Y	□	□	□	Varicella	□	□	□
Meningococcus B	□	□	□	Measles,mumps,rubella	□	□	□
Papillomavirus (HPV)	□	□	□	Influenza	□	□	□

B2. Influenza is a transmissible disease. Do you know how is it transmitted? (**more than one answer allowed**)
□ droplets □ foods and drinks □ contact with animals □ contact with contaminated surfaces □ uncertain □ other, specify______________________________

B3. Do you know how can influenza be prevented? (**more than one answer allowed**)
□ avoiding crowded places □ frequent disinfection of surfaces □ frequent handwashing □ room ventilation □ using masks □ keeping the distance of at least 1 mt □ avoiding touching one's face □ vaccination □uncertain □other, specify____

B4. In Italy, influenza vaccination is recommended to: (**more than one answer allowed)**
□ elderly □ health workers □ pregnant women □ healthy 6 months- 6 years old children □ persons of all ages with chronic conditions □ other, specify ________________________

C. Attitudes and behaviors towards influenza and related vaccination

C1. Please indicate whether you agree, disagree or are uncertain with the following statements:

	Agree	Uncertain	Disagree
It is better for children to acquire immunization through illness than through the vaccination	□	□	□
There are too many vaccinations for children	□	□	□

I am confident on the information about vaccinations for my child provided by my physician	□	□	□
Influenza vaccination protects against severe complications	□	□	□

C2. On a 1 to 10 scale, how much are you concerned that your child can develop influenza?

NOT AT ALL WORRIED 1 2 3 4 5 6 7 8 9 10 EXTREMELY WORRIED

C3. On a 1 to 10 scale, how much are you worried that the Influenza could have serious effects on your child's health?

NOT AT ALL WORRIED 1 2 3 4 5 6 7 8 9 10 EXTREMELY WORRIED

C4. On a 1 to 10 scale, how much is vaccination useful to prevent influenza in children?

NOT AT ALL USEFUL 1 2 3 4 5 6 7 8 9 10 EXTREMELY USEFUL

C5. Please indicate which of the following vaccination(s) has/have been uptaken by your child:
□ diphtheria □ tetanus □ pertussis □ poliomyelitis □ measles □ mumps □ rubella □ varicella □ meningococcus ACYW □ influenza

C6. Has your child ever received influenza vaccination? □ no □ yes, when? □ 2018\19 influenza seasons □ 2019\20 influenza seasons □ 2020\21 influenza seasons □ 2021\22 influenza seasons

C7. Do you intend to vaccinate your child in the upcoming 2022\2023 influenza season?

□ No, why? (**more than one answer allowed**)	□ Yes, why? (**more than one answer allowed**)
□ The vaccine is not safe<br>□ The vaccine is not effective<br>□ I am against vaccinations<br>□ Not recommended by pediatrician/ primary care physician<br>□ The influenza vaccine contains heavy metals<br>□ The vaccine can cause serious side effects<br>□ My child is not at risk<br>□ I do not trust institutions	□ The vaccine is safe<br>□ The vaccine is effective<br>□ I am favorable to vaccinations<br>□ It has been recommended by pediatrician/ primary care physician<br>□ To protect frail subjects<br>□ To distinguish influenza symptoms from those of COVID-19<br>□ My child is at risk<br>□ I trust institutions

D. Sources of information

D1. What are your sources of information about vaccinations? (**more than one answer allowed**)
□ none □ physician □ friends, relatives □ school □ tv, newspapers □ internet □ other, specify_____

D2. Do you feel you need more information about vaccination? □ no **(Stop)** □ yes

D3. Whom would you prefer to receive information from? (**more than one answer allowed**) □ friends, relatives □ physician □ school □ tv, newspapers □ other, specify_______________

Thank you for your participation to the survey!
